# The *ilv2* gene, encoding acetolactate synthase for branched chain amino acid biosynthesis, is required for plant pathogenicity by *Leptosphaeria maculans*

**DOI:** 10.1007/s11033-024-09620-4

**Published:** 2024-05-25

**Authors:** Nicholas F. Chong, Angela P. Van de Wouw, Alexander Idnurm

**Affiliations:** School of BioSciences, The University of Melbourne, Melbourne, VIC 3010 Australia

**Keywords:** Acetohydroxyacid synthase, *Brassica napus*, Chemical control, Pathogenicity, Phytopathogen

## Abstract

**Background:**

Control of blackleg disease of canola caused by the fungus *Leptosphaeria maculans* relies on strategies such as the inhibition of growth with fungicides. However, other chemicals are used during canola cultivation, including fertilizers and herbicides. There is widespread use of herbicides that target the acetolactate synthase (ALS) enzyme involved in branched chain amino acid synthesis and low levels of these amino acids within leaves of *Brassica* species. In *L. maculans* the *ilv2* gene encodes ALS and thus ALS-inhibiting herbicides may inadvertently impact the fungus.

**Methods and results:**

Here, the impact of a commercial herbicide targeting ALS and mutation of the homologous *ilv2* gene in *L. maculans* was explored. Exposure to herbicide had limited impact on growth in vitro but reduced lesion sizes in plant disease experiments. Furthermore, the mutation of the *ilv2* gene via CRISPR-Cas9 gene editing rendered the fungus non-pathogenic.

**Conclusion:**

Herbicide applications can influence disease outcome, but likely to a minor extent.

## Introduction

The ability of microbes to grow within their hosts relies on multiple strategies, one of which can be the *de novo* synthesis of metabolites. This can be vital to providing the ability to grow in environments with low concentrations of metabolites that may be of limited availability within the host, such as amino acids.

The *ilv2* gene encodes acetolactate synthase (ALS, or acetohydroxyacid synthase AHAS), the first enzyme in the biosynthesis of the branched chain amino acids (BCAAs) isoleucine, leucine and valine [1]. Making up three of the nine essential amino acids for humans, BCAAs are synthesized by plants, algae, fungi, bacteria, and archaea, but not animals [2, 3]. As such, *ilv2* and other enzymes of the BCAA biosynthesis pathway are targets for herbicides, fungicides, and other antimicrobials. There is considerable interest in acetolactate synthase inhibitors as new antifungals, illustrated by research on their potential control of human pathogenic fungi [4–9].

*Leptosphaeria maculans* is an ascomycete fungal plant pathogen that causes blackleg disease on *Brassica* crops, most notably canola (*Brassica napus*). *L. maculans* is the primary biotic limitation impacting canola production, e.g. in Australia causing average yield losses of 20–30% per annum [10] and representing a loss of 1–2 billion dollars per annum [11]. Additionally, canola is an essential part of the crop rotation system used to ensure high productivity for grain crops like wheat and barley, with one such application being in weed control.

Chemicals that inhibit ALS such as the Clearfield® herbicides are currently registered and in use for weed control when applied to canola cultivars that are resistant to them, due to a point mutation in the gene encoding ALS [12, 13]. Additionally, canola plants have very low levels of BCAAs in their leaves [14, 15], suggesting that *L. maculans* would typically have to make these amino acids to remain viable and cause disease in leaves. This could mean that already available chemicals that are intended to target plants may inhibit *L. maculans* inadvertently yet advantageously for canola production. This research into herbicide impacts and the *ilv2* gene aimed to test this potential unintended use of ALS-inhibiting herbicides as fungicidal agents in blackleg disease.

## Materials and methods

### Genetic manipulation of *L. maculans*

The *ilv2* gene was mutated through CRISPR-Cas9 based gene disruption, as outlined in *L. maculans* by Chambers et al. [16]. PCR was used to generate an amplicon from the oligonucleotide NC001 that includes 20 nucleotides to target *ilv2* with primers MAI0310 and MAI0556 (primer sequences are provided in Table 1). The PCR product was precipitated, then cloned into plasmid pMAI105, containing the CRISPR-Cas9 machinery, linearized with restriction enzyme NcoI, using the NEBuilder® Hifi DNA Assembly kit (New England Biolabs®). The assembled DNA was transformed into chemically-competent *Escherichia coli* cells by heat shock, then plasmid DNA was extracted and transformed by electroporation into *Agrobacterium tumefaciens* strain EHA105. The transfer DNA, that features the Cas9 endonuclease and guide RNA to target *ilv2*, was introduced into *L. maculans* strain D22 through *Agrobacterium*-mediated transformation with selection using hygromycin [17].

**Table 1:** Primers used in this study

Name	Sequence (5´-3´)	Purpose
NC001	ATCAACAACTTCATCTGCCTTTGCGCTGATGAGTCCGTGAGGACGAAACGAGTAAGCTCGTCCGCAAACTTGGCAATGGATCGTTTTAGAGCTAGAAATAGC	*ilv2* guide RNA oligonucleotides
NC007	CTGAACTCCTCAAGGAGC	Screening for CRISPR mutation in *ilv2*
NC008	AACCACTCTGGTCTCTCC	
NC024	TCCCAGAATTCTTAATTAAGATCGTTGTAGTTGTAGTAGC	*ilv2* complementation
NC025	TAGGCCTCTGCAGGTCGACTCTATCATCACCACTTGCG	
MAI0310	ATTTTAACTTGCTATTTCTAGCTCTAAAAC	Guide RNA construct
MAI0556	ATTTTAACTTGCTATTTCTAGCTCTAAAAC	

DNA was extracted from transformants, and a PCR using primers NC007 and NC008 amplified a region surrounding the site targeted for cleavage by the guide RNA. These samples were then digested with restriction enzyme BamHI, with the rationale that strains which had been cleaved by the CRISPR machinery would likely have a mutation in the restriction enzyme cut-site due to inaccuracies during DNA repair to have a different digest pattern to wild-type strains. The amplicons from one strain with a digest pattern different to wild-type were sequenced.

The *ilv2* mutant was complemented with the wild-type copy of the *ilv2* gene from *Leptosphaeria biglobosa*, taxonomically the closest relative of *L. maculans* [18]. The rationale for using this version of *ilv2* is that there is divergence in its sequence such that the CRISPR-Cas9 machinery, still present in the *ilv2* mutant strain, should not be able to target it. That is, in *L. maculans* the relevant 23 nucleotides are CGCAAACTTGGCAATGGATCCGG, where the first 20 nucleotides are guide RNA to direct Cas9 to cut the *ilv2* gene DNA, and the final cgg is the protospacer adjacent motif (PAM) site which is necessary for targeting the Cas9 machinery. The equivalent *L. biglobosa* sequence is CGCAAA*T*TTGGCAATGGC*GC*C*A*G, where the differences compared to *L. maculans* include three nucleotide changes to the guide RNA sequence and a change to the PAM site, as in italics. The *ilv2* gene was amplified from genomic DNA of *L. biglobosa* strain 06J154 [19] with primers NC024 and NC025. The PCR amplicon was gel-extracted and assembled into plasmid pMAI2 linearized with EcoRV and XbaI, using the NEBuilder Hifi DNA Assembly kit. The reaction products were then transformed into *E. coli* using heat shock. Plasmid DNA was extracted from *E. coli* colonies. A pool of plasmids was prepared by mixing 5 µl of plasmid DNA extracted from six colonies. This mix was then transformed into *Agrobacterium*, and subsequently the T-DNA carrying *ilv2* and a region conferring resistance to G418 into *L. maculans.*

### *Leptosphaeria maculans* pathogenicity assays

Seeds of *Brassica napus* (canola) Clearfield® cultivars Hyola® 580CT, Banker CL and Saintly CL, or Westar were sown in potting mix. Approximately 9 days after seed germination, the canola plants were inoculated with spore solutions of each of the *L. maculans* strains. This included the wild-type D22 strain [20], the mutant with gene-edited *ilv2* (termed ‘*ilv2* mutant’ from herein), and the complementation control strain (termed ‘*ilv2* complement’ from herein). Spore solutions were prepared by scraping pycnidiospores off sporulating cultures into sterile water. The solutions were filtered through Miracloth to remove agar and mycelia. Spores were washed twice with sterile water, and diluted to approximately 10^6^ spores/ml. Both lobes of the canola cotyledons were wounded with a sterile hypodermic needle, and 5 µl of spore solution was pipetted onto the wounded site. 8–10 plants were inoculated for each condition.

To test the effects of ALS inhibitors, the Clearfield® herbicide Intervix® was applied to plants at three different time points, on the same day as fungal inoculation with strain D22, three days post-infection, and five days post-infection. 10 µl of Intervix® solution (10 µl/ml) was applied directly around the plant wound as small droplets. Disease was assessed 14 days post-infection, with lesions photographed and sizes measured using ImageJ. Data analysis was performed in RStudio. To determine the average lesion size caused by the disease, an ‘equivalent circle radius’ was calculated. This involved taking the surface area of each lesion, and calculating a theoretical circle with an equal area. The radii of the circles were then used for statistical analyses to compare treatments. Two sample t-tests assuming unequal variance were performed with a statistical significance level of *p* = 0.05.

### Isoleucine, leucine, and valine auxotrophy assay

To determine the impacts of mutation in the *ilv2* gene on fungal growth, an auxotrophy assay was performed. Spore solutions of approximately equal concentration were prepared as described previously of the *ilv2* mutant strain, the *ilv2* complemented strain, and a wild-type strain. 5 µl of spore solution was pipetted into liquid Gamborg’s minimal media cultures, without the addition of amino acids or supplemented with isoleucine, leucine, and valine. Three cultures for each condition were grown at 22 °C for 28 days, before being freeze-dried and weighed.

### In vitro inhibition assays

HPLC-grade chemicals imazapyr, imazamox, and chlorimuron ethyl were purchased from Sigma-Aldrich, and dissolved in DMSO. Gamborg’s minimal media plates with increasing concentrations from 0.5 µg/ml to 256 µg/ml of each chemical were prepared. Three replicate cultures were prepared for each of D22, the *ilv2* mutant, and the *ilv2* complemented strain at each concentration. Equal-sized mycelial plugs were placed in the center of each plate. Following 10 days of growth at 22 °C, colony diameter measurements were taken on three horizontal axes on each plate.

## Results

### Creation of an *ilv2* mutant of *L. maculans*

**Fig. 1:**
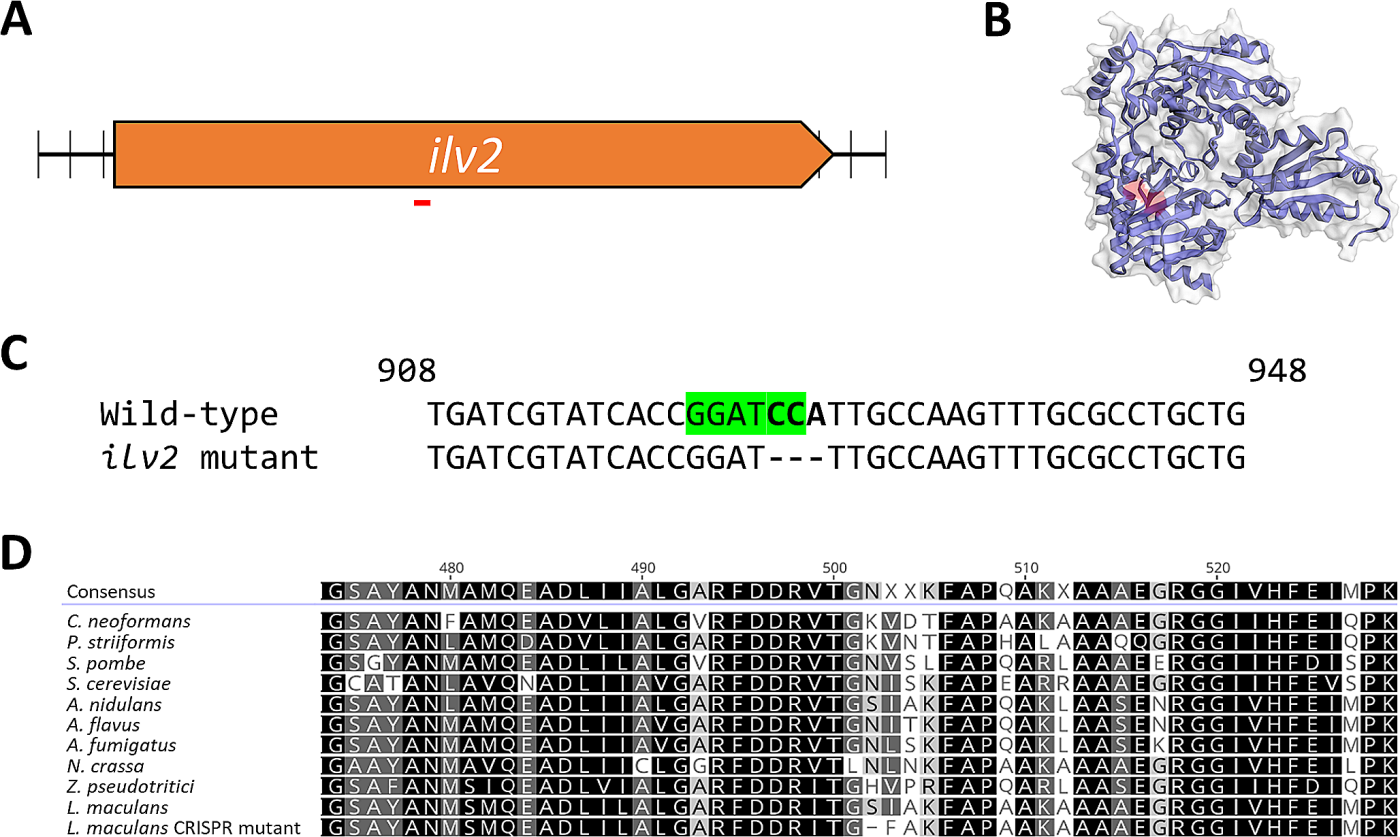
(**A**) Diagram of the *ilv2* gene with the target site for CRISPR-Cas9 mutation underlined. (**B**) Predicted 3D structure of the *L. maculans* Ilv2 protein with the mutated region highlighted in red. (**C**) Sanger sequencing data comparing the wild-type *ilv2* gene with the *ilv2* gene in the mutated strain. There is a 3 base-pair deletion in the mutant strain (bold font). The BamHI restriction enzyme site used for screening is highlighted in green. (**D**) Alignment of part of Ilv2 proteins with the *L. maculans* homolog from other fungi. The CRISPR mutation impacted two amino acids compared to the *L. maculans* wild-type, resulting in the loss of one amino acid and a replacement of the other. One of which (amino acid 503) is conserved as I, L, or V in fungi, however is mutated into a phenylalanine (**F**) in the CRISPR mutant

To test the role of *ilv2* on plant pathogenicity in *L. maculans*, an *ilv2*-disrupted mutant was generated by gene editing. The gene was identified in the *L. maculans* genome sequence by BLAST using the homolog from *Saccharomyces cerevisiae*. A guide RNA was designed to mutate a region within the middle of the gene with Cas9, and transformants were screened by PCR and the loss of the ggatcc restriction enzyme site. As seen in Fig. 1, a mutant with a three nucleotide deletion in the *ilv2* gene was generated. The predicted impact of this mutation on the protein is a loss of an amino acid and a change in one other amino acid.

### The *ilv2* mutant strain was unable to cause disease on canola

**Fig. 2:**
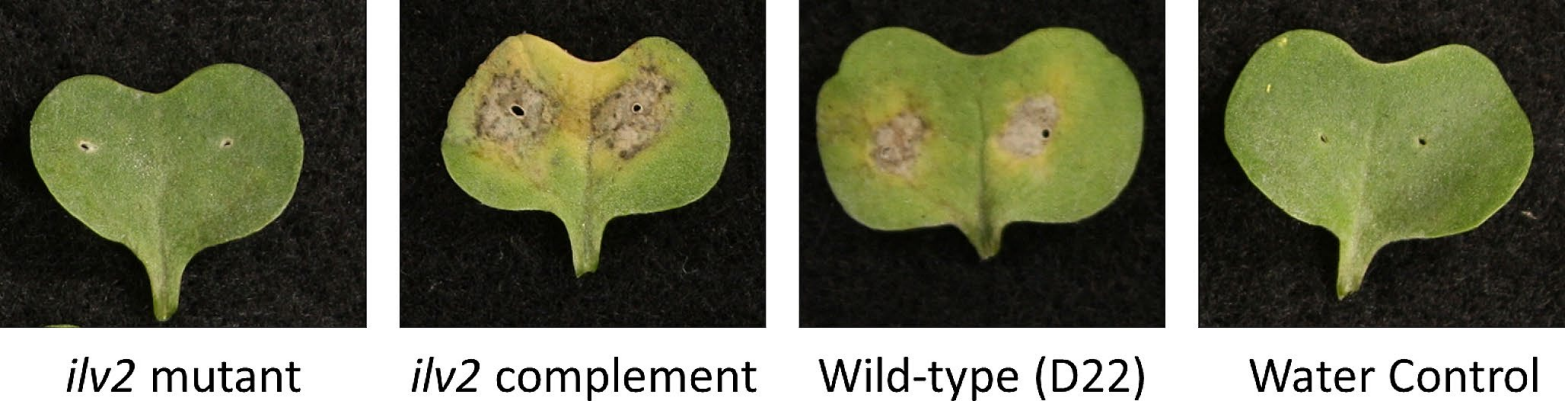
Representative images of canola cotyledons 21 days post-infection. Disease symptoms are visible for the wild-type strain (D22) as well as the *ilv2* complemented strain. Lesions are not present for the cotyledons infected with the *ilv2* CRISPR mutant strain or water control

To determine whether *ilv2* impacted the ability of *L. maculans* to cause disease on canola, a plant disease assay was performed. The canola variety Westar was used due to its high susceptibility to *L. maculans*. As shown in Fig. 2, plant disease symptoms were not visible for the *ilv2* mutant, more similar to the water control treatment than the plants infected with wild-type strain D22. The wild-type copy of *ilv2* from *L. biglobosa* was transformed into the mutant strain, and two transformants selected on media containing G418. The complemented strains caused similar lesions as wild-type (Fig. 2).

### ALS-inhibiting herbicides impact *L. maculans* pathogenicity on canola

**Fig. 3:**
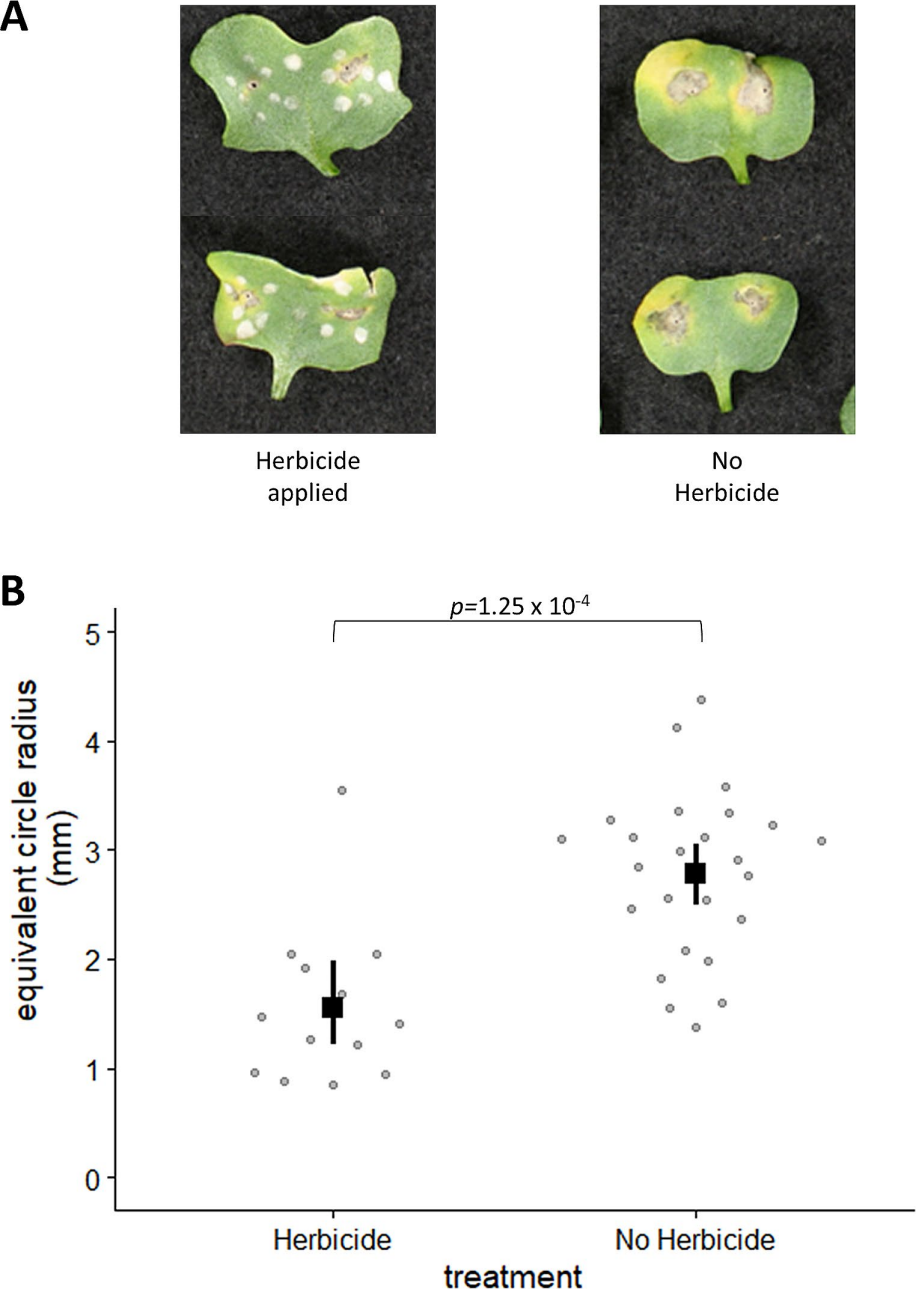
(**A**) Representative images of canola cotyledons 14 days post-infection. Intervix® herbicide was applied three days post inoculation. Herbicide-treated cotyledons displayed small characteristic *L. maculans* necrotic lesions surrounding the point of infection, as well as chlorotic patches across the leaf. Plants without exposure to herbicides showed standard-sized necrotic lesions around the point of infection. (**B**) Graph showing the average lesion size for the herbicide-treated and untreated plants, through calculation of the equivalent circle radius with 95% confidence intervals as shown by the black lines. There is a clear distinction between the confidence intervals, with no overlapping sections. Further, a *p*-value of 1.25 × 10^− 4^ was generated using a two-sample t-test (assuming unequal variance)

To assess whether the *ilv2* product, ALS, could be chemically inhibited during plant pathogenesis by *L. maculans*, the herbicide Intervix® that functions by inhibiting plant ALS was tested for its pathogenicity impacts. Lesion sizes in Clearfield® plants, which are resistant to Intervix®, treated with herbicide were significantly smaller than those in the control treatment (Fig. 3).

**Fig. 4:**
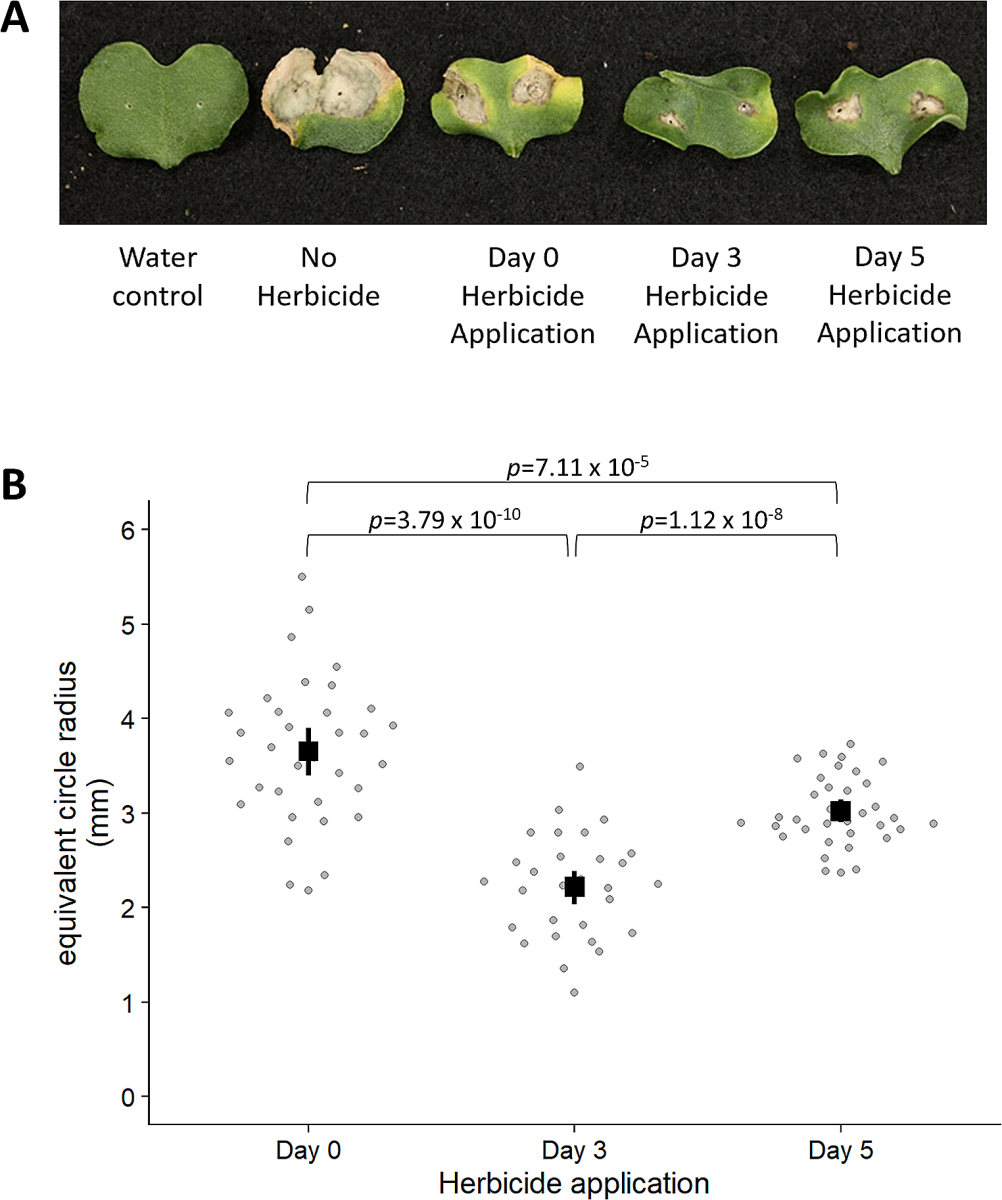
(**A**) Representative canola cotyledons depicting the effect of herbicide application timing on disease symptoms. Plants that were not treated with herbicide displayed the highest level of disease symptoms and largest lesions; indeed so many cotyledons had extensive lesions and/or fell off the plants that these were not included in the statistical analyses. (**B**) Graph showing the average lesion size for the herbicide-treated and untreated plants, through calculation of the equivalent circle radius with 95% confidence intervals as shown by the black lines. All plants treated with herbicide had smaller lesions, with the plants being treated 3 days post-inoculation having the smallest average lesion size compared to plants treated on the same day as inoculation (day 0) and 5 days post-inoculation. Confidence intervals between groups have no overlaps. *P*-values were generated between groups, with a *p*-value of 3.79 × 10^− 10^ between the day 0 and day 3 conditions, 7.11 × 10^− 5^ between day 0 and day 5, and a *p*-value of 1.12 × 10^− 8^ between day 3 and day 5

The timing of herbicide application relative to disease inoculation was tested. Lesion sizes in plants treated with the herbicide Intervix® 3 days after inoculation were smaller than those which were treated on the same day as inoculation and 5 days after inoculation (Fig. 4).

### The *L. maculans ilv2* mutant did not have distinct growth differences when grown in vitro on media containing isoleucine, leucine, and valine

**Fig. 5:**
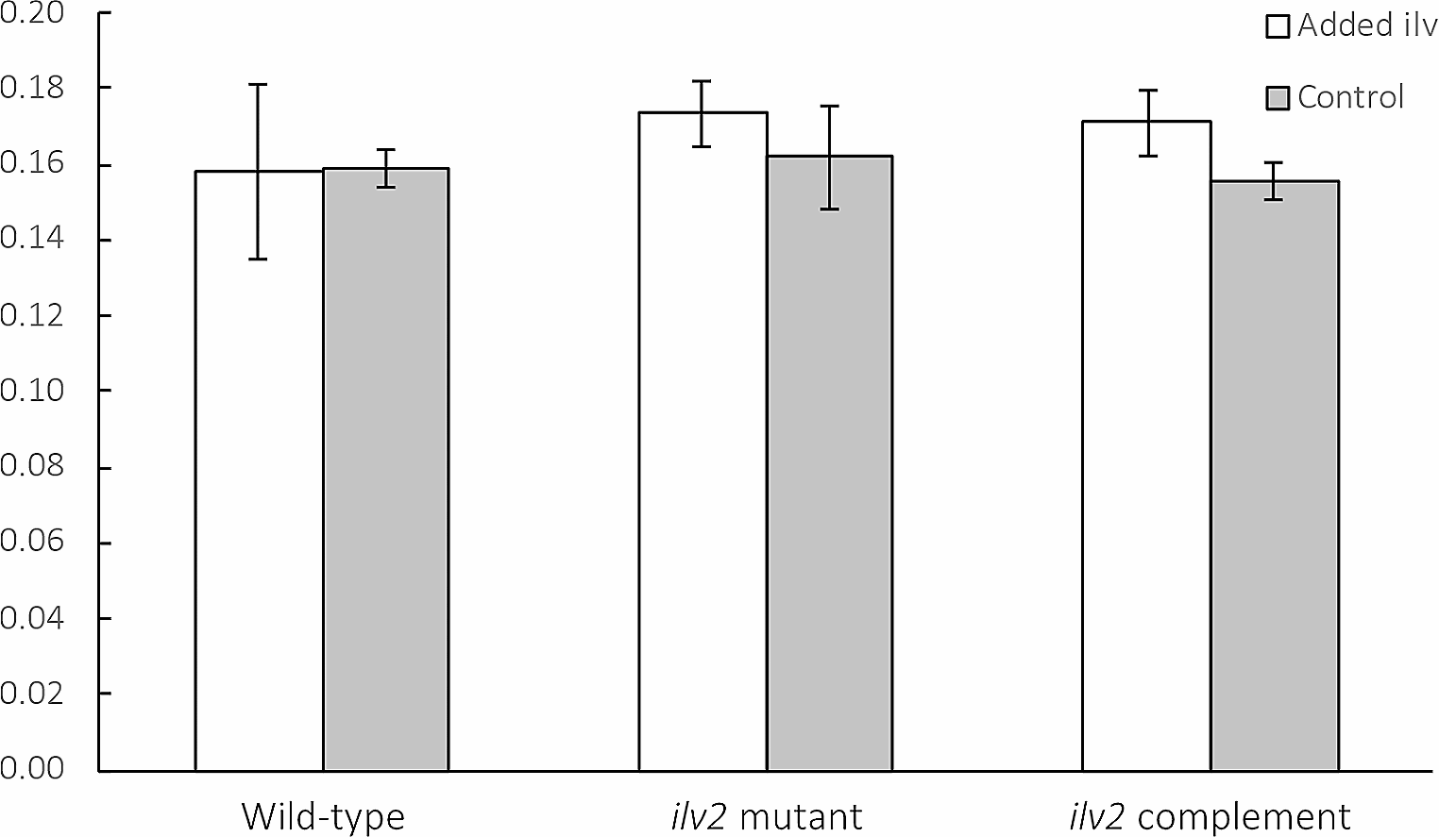
Grouped bar chart depicting the average biomass for each strain when grown in Gamborg’s minimal media with added isoleucine, leucine, and valine, and Gamborg’s minimal media without any supplements. Error bars representing the standard error are shown. No strain showed a distinct difference in growth between the BCAA-supplemented and standard minimal media

The impact of supplemental BCAAs on growth for the *L. maculans ilv2* mutant strain was tested by measuring the biomass after 28 days of growth in liquid media. The wild-type, the *ilv2* mutant, and the *ilv2* complemented strain showed little growth difference between conditions with and without supplemental BCAAs where biomass was used as a measurement rather than radial colony growth due to sporadic growth patterns observed on minimal media (Fig. 5).

### In vitro growth inhibition did not occur in *L. maculans* strains on media plates supplemented with imazapyr and imazamox, but did occur in plates supplemented with chlorimuron ethyl

**Fig. 6:**
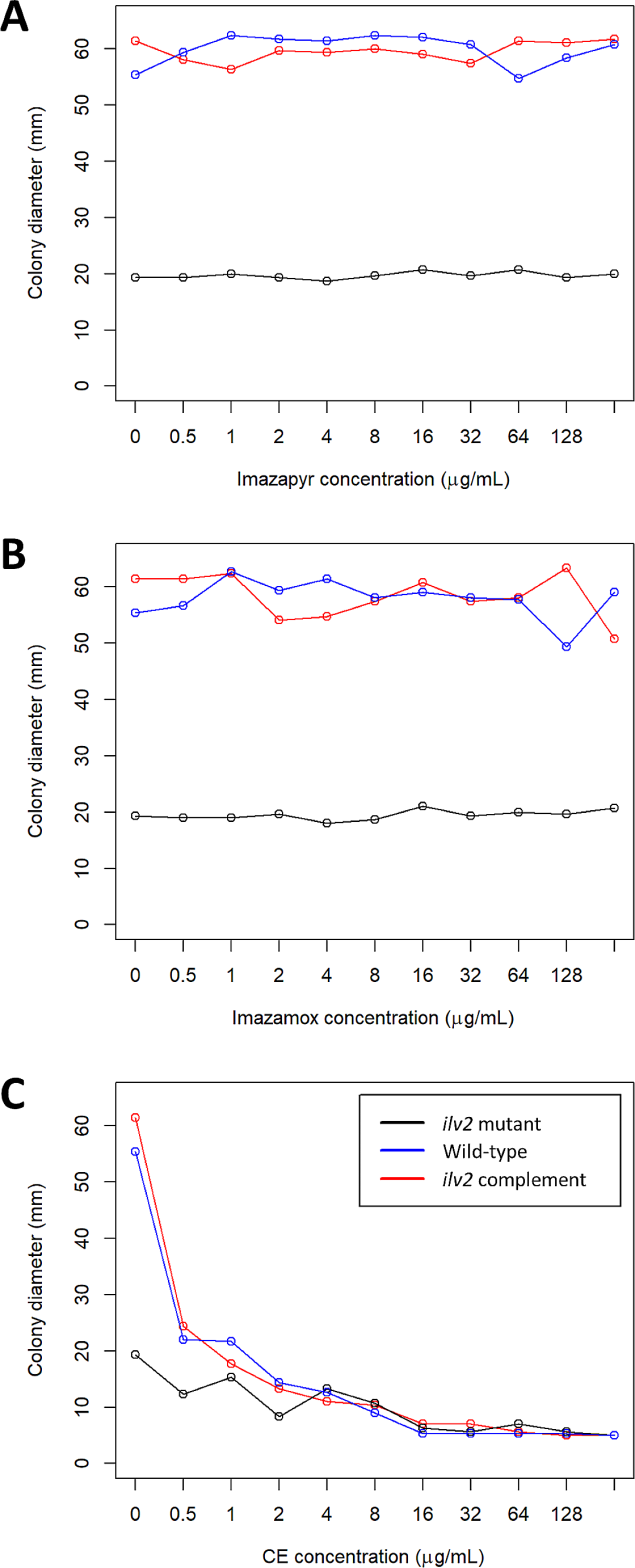
Graphs showing the colony diameter (average of 3 measurements per plate) for each chemical concentration. (**A**) Each strain grown on imazapyr was largely unaffected by the chemical up to 256 µg/ml. (**B**) Each strain grown on imazamox was similarly unaffected by the presence of imazamox up to 256 µg/ml. (**C**) When grown on chlorimuron ethyl, each strain displayed growth inhibition with as little concentration as 0.5 µg/ml

Three *L. maculans* strains were grown on plates supplemented with three ALS inhibitory chemicals, i.e. imazapyr, imazamox, and chlorimuron ethyl, to determine the in vitro effects of herbicidal chemicals on *L. maculans* growth. Intervix® is a mixture of both imazapyr and imazamox, and neither of these applied individually altered growth of these strains up to 256 µg/ml (Fig. 6A and B). Chlorimuron ethyl, which is particularly effective at inhibiting human pathogenic fungi, greatly reduced *L. maculans* growth, even at a concentration as low as 0.5 µg/ml (Fig. 6C).

## Discussion

The *ilv2* mutant of *L. maculans* was unable to infect canola plants. Reduced pathogenicity of *ilv2* mutants has been observed in other fungi. In the human pathogens *Cryptococcus neoformans* [21] and *Candida albicans* [22] *ilv2* mutants lost viability in the absence of supplemental isoleucine and valine, and could not survive or cause disease in mice. Further, use of herbicides that inhibit ALS have successfully treated *C. albicans-*infected mice [5].

The *ilv2* mutant strain was slow-growing (Fig. 6) and those growth defects could be an explanation of the inability of the mutant to cause plant disease. That is, the mutant may have general viability issues which impact its ability to cause disease on plants. This was noted by Liu et al. [23] in *Fusarium graminearum*, where it was difficult to separate reduced or lost plant virulence from the in vitro phenotypic defects of *ilv2* or *ilv6* mutants. They suggested that the limited ability of the fungus to acquire branched chain amino acids from the host during infection partially explains the reduced pathogenicity: branched chain amino acids are naturally low in abundance in canola [14], potentially requiring *L. maculans* to synthesize these amino acids.

The timing of herbicide application may factor into susceptibility to disease. Murtza et al. [24] tested the impacts of the herbicides glyphosate and atrazine on canola susceptibility to the foliar fungal diseases *Neopseudocercosporella capsellae*, *Alternaria brassicae, A. japonica*, and *Hyaloperonospora brassicae.* They found that the timing of the herbicide application impacted the fungal pathogen disease severity and incidence. Further, the impacts and timings varied between pathogens and herbicides. This study suggests that disease caused by *L. maculans* may also be affected by herbicide application timing (Fig. 4), whereby plants treated with Intervix® 3 days post-infection have less disease than those treated on the day of infection, and 5 days post-infection. When used in the field, Intervix® is sprayed on Clearfield® canola seedlings at the 2–6 leaf stage of their development [25]. This matches the timing for fungicide use for seedling protection [26], with registered fungicides such as Prosaro®, Veritas®, and Proviso® having label application timings at the 4–6 leaf stage.

Plants infected with *L. maculans* and then treated with Intervix® had smaller lesion sizes than untreated plants (Fig. 3). This supports the loss of pathogenicity caused by mutation of *ilv2*. Curiously, however, we saw limited impact of the individual components in this herbicide on fungal growth in vitro (Fig. 6). The reason for this is unclear: one hypothesis is that there is some residual activity of the *ilv2* gene considering the nature of the mutation only impacting two amino acids (Fig. 1D). We also explored the impact of chlorimuron ethyl, as previous research examining the impact of different ALS inhibitors on fungi found a range of responses, with chlorimuron ethyl being particularly effective against human pathogens [5]. Chlorimuron ethyl did inhibit growth of *L. maculans* in vitro, a finding that suggests it may be possible to find an ALS inhibitor with dual functions in the control of weeds and as an antifungal agent, repurposing pre-existing approved herbicides, such as Intervix®, as an additional measure to control *L. maculans* in the field. However, chlorimuron ethyl is toxic to plants, including the Clearfield® lines.

Another approach to apply this knowledge to an agricultural setting would be through host-induced gene silencing, which would involve engineering canola varieties that target the *L. maculans ilv2* mRNA. This was performed in cotton to resist Verticillium wilt caused by *Verticillium dahliae* [27], where ALS is required for pathogenicity [28]. Wei et al. [27] found that engineered expression of artificial RNAs in plants could reduce the transcript levels of *ilv2* and *ilv6* to inhibit disease symptoms in cotton. Host-induced gene silencing technology is still in its infancy, however, and has several limitations. This includes observations that silencing efficiency by this method varies considerably and lacks consistency [29–31], and it also requires transformation of plants, which can be an issue with public acceptance and governmental regulations of genetically-modified organisms [32].

## Data Availability

Data is provided within the manuscript.
